# Clinical efficacy and safety of linezolid in intensive care unit patients

**DOI:** 10.1016/j.jointm.2022.05.006

**Published:** 2022-07-05

**Authors:** Aijia Ma, Meiling Dong, Jiangli Cheng, Xuelian Liao, Wei Dong, Chang Liu, Chenggong Hu, Jing Yang, Yan Kang

**Affiliations:** Department of Critical Care Medicine, West China Hospital of Sichuan University, Chengdu, Sichuan 610041, China

**Keywords:** Intensive care unit, Linezolid, Real-world study, Gram-positive bacterial infection

## Abstract

**Background:**

To characterize the population of critically ill patients and infections treated with linezolid in the intensive care unit (ICU), and to evaluate the clinical efficacy and safety of linezolid therapy.

**Methods:**

This multi-center, observational, real-world study was conducted across 52 hospitals between June 9, 2018, and December 28, 2019. Patients who met the following inclusion criteria were included: (1) admitted to the ICU, (2) of any age group, and (3) having a clinical or laboratory diagnosis of a Gram-positive bacterial infection. Clinical efficacy was categorized as success (cured or improved), failed, or non-evaluable. Adverse events and serious adverse events were recorded during treatment.

**Results:**

A total of 366 ICU patients who met the inclusion criteria were evaluated. Linezolid was used as second- and first-line treatment in 232 (63.4%) and 134 (36.6%) patients, respectively. The most common isolated strain was *Staphylococcus aureus* (methicillin-resistant *Staphylococcus aureus: n*=37/119, 31.1%; methicillin-susceptible *Staphylococcus aureus: n*=15/119, 12.6%); this was followed by *Enterococci* (vancomycin-resistant *Enterococci: n*=8/119, 6.7%; vancomycin-susceptible *Enterococci: n*=11/119, 9.2%) and *Streptococcus pneumoniae* (multidrug-resistant: *n*=4/119, 3.4%; non-multidrug resistant: *n*=2/119, 1.7%). The main infection sites where pathogens were detected included the lung (*n*=216/366, 59.6%), skin and soft tissue (*n*=104/366, 28.4%), and blood (*n*=50/366, 13.7%). Clinical success was achieved in 301 (82.2%) patients; 34 (9.3%) were cured and 267 (73.0%) improved; treatment failure and non-evaluable outcomes were observed in 29 (7.9%) in 36 (9.8%) patients, respectively. Linezolid-related adverse events were reported in 8 (2.2%) patients. No treatment-related serious adverse events were reported.

**Conclusions:**

Based on real-world results, linezolid was found to be effective and safe in the treatment of Gram-positive bacterial infections in critically ill patients.

## Introduction

The intensive care unit (ICU) is the “hardest hit” department for hospital-acquired infections, where a range of traumatic procedures can lead to fatal infections.^[^^1^^,^^2^^]^ Ventilator-related pneumonia, catheter-related bloodstream infections caused by an arterial or venous puncture, and skin and soft tissue infections (SSTIs) secondary to tracheotomy and surgical wounds are the main types of hospital-acquired infections.[3], [4], [5] Nearly half of these infections are caused by Gram-positive bacteria.^[6]^ The increasing prevalence of multidrug-resistant Gram-positive pathogens poses a significant challenge in the ICU.^[7]^ Methicillin-resistant *Staphylococcus aureus* (MRSA), methicillin-susceptible *Staphylococcus aureus* (MSSA), and vancomycin-resistant *Enterococci* (VRE), which are extremely common in the ICU, are regarded as priority pathogens that cause morbidity and mortality in countless cases.^[^^6^^,^^8^^]^ Therefore, it is essential for ICU clinicians to identify resistance patterns in Gram-positive bacteria and use antibiotics that are effective against these resistant phenotypes.

Linezolid, a synthetic oxazolidinone antibiotic, has been approved for the treatment of infections caused by VRE, hospital-acquired pneumonia caused by MRSA and MSSA, complicated SSTIs caused by *Staphylococcus aureus* or *Streptococcus pneumoniae*, and uncomplicated SSTIs caused by *Staphylococcus aureus* (methicillin-resistant only) or *Streptococcus pyogenes*.^[9]^ The drug has favorable *in vitro* and *in vivo* activity against the mentioned organismsy^[^^10^^]^

As advanced antibiotics commonly used in the ICU, linezolid and vancomycin are often compared. Although vancomycin is often used as the first choice, side effects pertaining to liver and kidney function, and especially renal injury, lead to certain limitations in its use in ICU patients.^[11]^ Linezolid is more useful than vancomycin in SSTIs and MRSA pneumonia,^[12]^ and is more effective and cost-effective for hospital-acquired MRSA infections.^[13]^ It is also the only antibiotic that is more effective than daptomycin and quinopidine (among others) for vancomycin-resistant *Enterococcus faecalis* infection.^[^^14^^,^^15^^]^

Considering the role of linezolid in the treatment of Gram-positive bacteria, this real-world study was conducted to characterize the population of critically ill patients in the ICU and infections treated with linezolid in the ICU. It also aimed to evaluate the clinical efficacy of linezolid therapy and to assess the safety of Chinese-made linezolid in ICU patients. This study collected data from 52 hospitals and conducted a retrospective analysis to guide better antibiotic prescribing among clinicians.

## Methods

### Patient population

This multi-center, observational, real-world study conducted across 52 hospitals between June 9, 2018 and December 28, 2019 was approved by the ethics committee of the West China Hospital, Sichuan University, as well as the institutional boards of the other 51 centers (No. 30/2017).

Patients consented to participate in the study and met the following inclusion criteria: (1) admitted to the ICU, (2) of any age group, and (3) having a clinical or laboratory diagnosis of a Gram-positive bacterial infection.

### Research methods

The research drug was linezolid injection (200 mg/100 mL) under the brand name Hengjie, which was produced by the Jiangsu Hansoh Pharmaceutical Group Co., Ltd. (H20150223). The start of linezolid injection was used as the start time point in the study; patients were followed up once a day until 48 h after the discontinuation of therapy, transfer out of the ICU, or death.

### Clinical efficacy

The primary efficacy indicators were categorized as clinically cured, clinically improved, clinically failed, or non-evaluable. Clinical cure was defined as the absence of signs and symptoms, the absence of subsequent antibiotic medication, or the absence of infection on a culture report. Clinical improvement was defined as the partial disappearance of clinical signs and symptoms and/or the need for more antibiotics. Patients who died or had an unsatisfactory response to linezolid therapy, worsening or recurring signs and symptoms, requirements for a change in antibiotic medication, or a positive culture at the end of therapy were considered to have clinically failed. Insufficient information making it impossible to determine a response led to categorization as non-evaluable.^[^^16^^,^^17^^]^ Clinical success included cured or improved categories.

Secondary efficacy indicators included organ function status, bacterial clearance rate, negative rate of pathogenic cultures, ICU stay time, duration of hospital stay, and ICU mortality and mortality.

### Safety

Adverse events (AEs) referred to adverse medical events that occurred after patients or clinical trial subjects received linezolid, but were not necessarily causally related to treatment. Serious adverse events (SAEs) were defined by meeting one or more of the following criteria: (1) death, (2) life-threatening complications, (3) necessitating hospitalization or prolonged hospitalization, and (4) permanent or severe disability.^[^^16^^,^^17^^]^ The researchers monitored AEs and SAEs for 30 days after receipt of the medication. The severity of all reported AEs was evaluated by local investigators, regardless of whether they were related to linezolid. The AEs and SAEs were categorized into five levels: definitely related, very likely to be related, possibly related, probably related, and unlikely or not related. The standard protocols for the identification and treatment of AEs were customized by clinicians according to the clinical condition.

### Combined therapy

The combined medication included two components, one being the antibiotic itself. As linezolid injection has no activity against Gram-negative bacteria, the investigator could add antibiotics as appropriate in clinically suspected or confirmed cases of Gram-negative bacterial infections. Antifungal drugs were used as appropriate when fungal infections were suspected or diagnosed. The other component was the main drug used for the treatment of the primary disease (if there were other major concomitant diseases). This study mainly evaluated whether linezolid injection demonstrated cross-reactivity with certain drugs.

### Data collection

Data were obtained from patients at 52 institutions using a standardized form and process. The data included the following: (1) demographic data; (2) clinical baseline data on the first day of medication including microbiological data and acute physiology and chronic health evaluation (APACHE II), sequential organ failure assessment (SOFA), and Glasgow coma scale (GCS) scores; (3) clinical effectiveness and safety data; and (4) pharmacoeconomic indicators including total costs of linezolid use, antibiotic use, and ICU stay.

### Statistical analysis

SPSS version 22.0 was used for statistical analysis. No inferential analyses were performed; only descriptive statistics were employed. All the analyses were explanatory. For continuous variables, numerical data were reported as means ± standard deviation, medians (interquartile range), and minimum and maximum. Absolute and relative frequencies were used to summarize categorical data.

## Results

### Patient demographics and clinical characteristics

A total of 366 ICU patients who met the inclusion criteria were evaluated. The demographic, clinical, and infection characteristics are shown in Table 1. There were 246 (67.2%) and 120 (32.8%) male and female patients, respectively. The mean age was 57.3 ± 22.9 years, and the majority (66.4%) of patients were over 65 years old. A total of 30 children were included in this study; they had an average age of 6.2 ± 5.9 years. The mean body weight of the cohort was 60.8 ± 17.4 kg, with a mean body mass index of 22.7 ± 3.9 kg/m^2^. Patients from different grade hospitals were well represented; the majority (89.9%) were derived from grade III level A hospitals.

**Table 1:** Demographic and clinical characteristics (*n=*366).

Characteristic	Patients	Characteristic	Patients
Hospital grade		G+ infection site	
Grade III level A hospital	329 (89.9)	Lung	216 (59.6)
Grade II level A hospital	26 (7.1)	SSTI	104 (28.4)
Grade II level B hospital	11 (3.0)	Blood	50 (13.7)
Gender		Lung + SSTI	62 (16.9)
Male	246 (67.2)	Lung + blood	13 (3.5)
Female	120 (32.8)	SSTI + blood	15 (4.1)
Age (years)	57.3 ± 22.9	Other	62 (16.9)
<18	30 (8.2)	Diagnosed of combined G− bacteria infection	199 (54.4)
18–65	90 (24.6)	Suspected of combined G− bacteria infection	156 (42.6)
>65	243 (66.4)	Diagnosed of complicated fungal infection	34 (9.3)
Body weight(kg)	60.8 ± 17.4	Suspected of complicated fungal infection	41 (11.2)
Body mass index(kg/m^2^)	22.7 ± 3.9	Types of skin infections	
Reasons for entering ICU		Simple (without fever)	23 (6.3)
Acute respiratory disease syndrome	28 (7.7)	Complexity (with fever)	343 (93.7)
Sepsis	12 (3.3)	APACHE II score	17.9 ± 8.3
Respiratory failure	134 (36.6)	GCS score	11.5 ± 3.5
Circulatory failure	127 (34.7)	SOFA score	6.4 ± 4.6
Respiratory and cardiac arrest	17 (4.6)	Physical therapy	132 (36.1)
Severe infection	169 (46.2)	Surgical intervention	51 (13.9)
Multiple trauma	32 (8.7)	Renal replacement therapy	25 (6.8)
Acute pancreatitis	15 (4.1)	Non-invasive ventilation	45 (12.3)
After high-risk surgery	48 (13.1)	Time of non-invasive ventilation (h)	71.8 ± 109.3
MODS	10 (2.7)	Cost of non-invasive ventilation (Yuan)	1407.1 ± 3305.0
Underlying diseases		Invasive ventilation	124 (33.9)
Hypertension	119 (32.5)	Time of invasive ventilation (h)	326.6 ± 371.9
Diabetes	61 (16.7)	Cost of non-invasive ventilation (Yuan)	7087.3 ± 20,199.2
COPD	45 (12.3)	ICU duration (days)	12.5 ± 11.6
Chronic heart failure	39 (10.7)	Daily dosage of linezolid (mg)	1109.9 ± 309.9
Coronary heart disease	8 (2.2)	Linezolid duration (days)	5.1 ± 4.9
Hematological diseases	14 (3.8)	Total cost of linezolid (Yuan)	3320.9 ± 1912.7
Tumor	27 (7.4)	Total antibiotic cost (Yuan)	8222.6 ± 7199.6
Chronic renal insufficiency	37 (10.1)	Total cost of ICU hospitalization (Yuan)	74,369.9 ± 84,623.3
Chronic kidney insufficiency	13 (3.6)	Clinical efficacy	
Immune system diseases	13 (3.6)	Clinical cured	267 (73.0)
Linezolid as second-line treatment	232 (63.4)	Clinical improved	34 (9.3)
Reasons for using linezolid		Clinical failure	29 (7.9)
Diagnosed G+ bacteria infection	119 (32.5)	Death	17 (4.6)
Suspected G+ bacteria infection	247 (67.5)	No response	12 (3.3)

Data are presented as *n* (%) and mean ± standard deviation.

APACHE II: Acute physiology and chronic health evaluation; COPD: Chronic obstructive pulmonary disease; G+: Gram-positive bacteria; GCS: Glasgow coma scale; ICU: Intensive care unit; MODS: Multiple organ dysfunction syndrome; SOFA: Sequential organ failure assessment; SSTI: Skin and soft tissue infections.

The included patients were from 9 ICUs, which mainly included comprehensive (53.3%), respiratory (18.6%), emergency (11.2%), pediatric (6.5%), cardiac (4.9%), and neurological (3.0%) ICUs. Severe infection (46.2%), respiratory failure (36.6%), and circulatory failure (34.7%) were the most common reasons for ICU admission. The significant underlying diseases were hypertension (32.5%), diabetes (16.7%), chronic obstructive pulmonary disease (12.3%), and chronic heart failure (10.7%).

In critically ill patients, the APACHE II score is a comprehensive score that allows assessment of the severity of the disease and predicts the risk of death. In this study, the mean APACHE II score of the entire cohort was 17.9 ± 8.3. The mean SOFA score, which evaluates organ function, was 6.4 ± 4.6. The GCS score is used to describe the extent of impaired consciousness in patients; the higher the score, the better the state of consciousness. The mean GCS score in the study cohort was 11.5 ± 3.5.

In total, 124 (33.9%) patients received invasive mechanical ventilation (via endotracheal intubation), and 45 (12.3%) patients received non-invasive mechanical ventilation for respiratory support. Overall, 25 (6.8%) patients received renal replacement therapy. During the trial, 132 (36.1%) patients received physical therapy for rehabilitation training and 51(13.9%) received surgical intervention.

### Microbiology

Among all included patients, linezolid was used in 119 (32.5%) patients after the isolated bacterial strain was diagnosed to be Gram-positive; in the remaining 247 (67.5%), antibiotics were prescribed empirically.

The most common isolated strain was *Staphylococcus aureus* (MRSA: *n*=37/119, 31.1%; MSSA: *n*=15/119, 12.6%); this was followed by *Enterococcus faecalis* (vancomycin-resistant: *n*=8/119, 6.7%; vancomycin-susceptible: *n*=11/119, 9.2%) and *Streptococcus pneumoniae* (multidrug resistant: *n*=4/119, 3.4%; non-multidrug resistant: *n*=2/119, 1.7%) (Table 2).

**Table 2:** Confirmed primary isolated pathogens in patients for whom culture results were obtained (*n=*119).

Pathogens	Patients
*Staphylococcus aureus*	
MRSA	37 (31.1)
MSSA	15 (12.6)
Other Staphylococcus*	15 (12.6)
*Enterococci*	
VRE	8 (6.7)
VSE	11 (9.2)
*Streptococcus pneumoniae*	
Multidrug-resistant	4 (3.4)
Non-multidrug-resistant	2 (1.7)
*Other Streptococcus*†	9 (4.5)
*Staphylococcus*	
*Mycobacterium tuberculosis*	1 (0.8)
*Corynebacterium*	
*Corynebacterium striatum*	1 (0.8)
Others	16 (13.4)

MRSA: Methicillin-resistant *Staphylococcus aureus*; MSSA: Methicillin-susceptible *Staphylococcus aureus*; VRE: Vancomycin-resistant *Enterococci*; VSE: Vancomycin-susceptible *Enterococci*.

⁎ Including *Staphylococcus hominis, Staphylococcus haemolyticus, Staphylococcus   epidermidis, Staphylococcus xylose, Staphylococcus haemophilus*, and *Staphylo-   coccus coriolis*.

† Including *Streptococcus viridans, Streptococcus constellation, Streptococcus pyo-   genes*, and *Candida albicans*.

Overall, 199/366 (54.4%) and 156/366 (42.6%) patients were diagnosed with and suspected to have combined Gram-negative bacterial infections, respectively. A total of 34/366 (9.3%) and 41 (11.2%) patients were diagnosed with and suspected to have combined complicated fungal infections, respectively.

**Figure 1:**
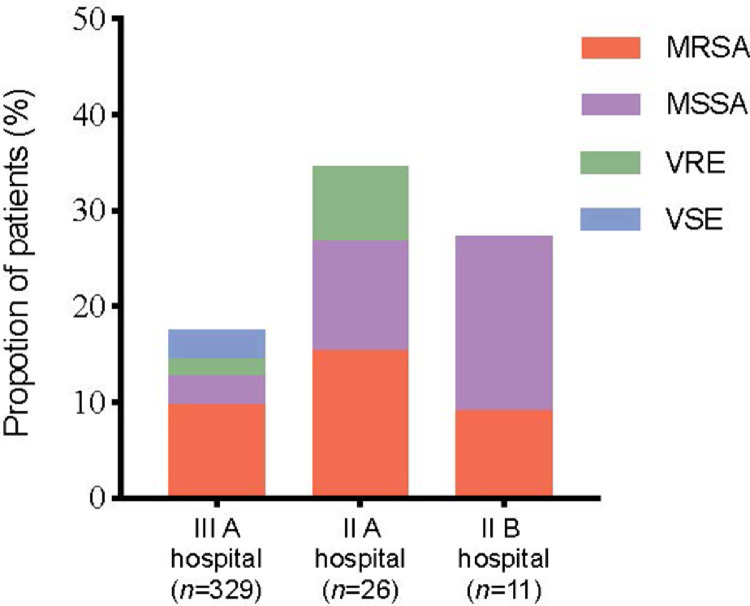
Proportion of different pathogens in hospitals. II A hospital: Grade II level A hospital; II B hospital: Grade II level B hospital; III A hospital: Grade III level A hospital; MRSA: Methicillin-resistant *Staphylococcus aureus*; MSSA: Methicillin-susceptible *Staphylococcus aureus*; VRE: Vancomycin-resistant *Enterococci*; VSE: Vancomycin-susceptible *Enterococci*.

The proportions of isolated strains in the three grades of hospitals are shown in Figure 1. MRSA accounted for the largest proportion in grade III level A and grade II level A hospitals.

### Infection sites

The main infection sites where pathogens were detected included the lungs (*n*=216/366, 59.6%), skin and soft tissues (*n*=104/366, 28.4%), and blood (*n*=50/366, 13.7%); pathogens were detected from the alveolar lavage fluid, secretions, pus from skin tissue, and blood. Most patients had multiple infection sites; these included the lung + skin and soft tissue (*n*=62/366, 16.9%), lung + blood (*n*=13/366, 3.5%), and skin and soft tissue + blood (*n*=15/366, 4.1%). Among patients with SSTI, most were identified as having complicated SSTI (*n*=343/366, 93.7%).

### Previous and combined antibiotic therapy

Overall, linezolid was used for second-line treatment in 232 (63.4%) patients. Most were treated with other antibiotics before initiation of linezolid therapy. The most common reason for stopping prior antibiotics was an antibiotic failure. Among those who had previously received vancomycin (*n*=207), 173 (83.6%) and 34 (16.4%) switched due to failure and intolerance, respectively.

During linezolid treatment, 251 (68.6%) patients received combined antibiotic therapy; these most often included carbapenems (*n*=143/251, 57.0%) and cephalosporins (*n*=41/251, 1.6%) (Table 3).

**Table 3:** Combined antibiotics (*n=*251).

Drug name	Cases	Drug name	Cases
Cephalosporins		Penicillins/ β-lactamase inhibitors	
Cefotaxime	1	Mezlocillin sulbactam	1
Cefoxitin	1	Piperacillin sulbactam	17
Ceftizoxime	4	Cefoperazone sulbactam	33
Ceftazidime	16	Carbapenems	
Ceftriaxone	3	Meropenem	63
Cefminox	6	Imipenem	54
Laoxycephalosporin	10	Biapenem	25
Quinolones		Ertapenem	1
Moxifloxacin	3	Antifungal drugs	
Levofloxacin	7	Caspofungin	6
Ciprofloxacin	1	Vulikangcuo	9
Aminoglycosides		Ornith file	2
Etimicin	1	Omeprazole	4
Amikacin	2		
Tetracyclines			
Tigecycline	7		

Co-administration of two drugs may alter their effectiveness. This interaction may delay, decrease, or increase the absorption of the drug, or cause adverse reactions. In this study, we did not observe any adverse effects when linezolid was co-administered with Gram-negative antibiotics, namely, cephalosporins, ciprofloxacin, meropenem, and gentamicin. In addition, the use of linezolid with antifungal drugs such as aminoglycosides and fluoroquinolones did not appear to affect the effectiveness of the either drug.

### Clinical effectiveness

**Figure 2:**
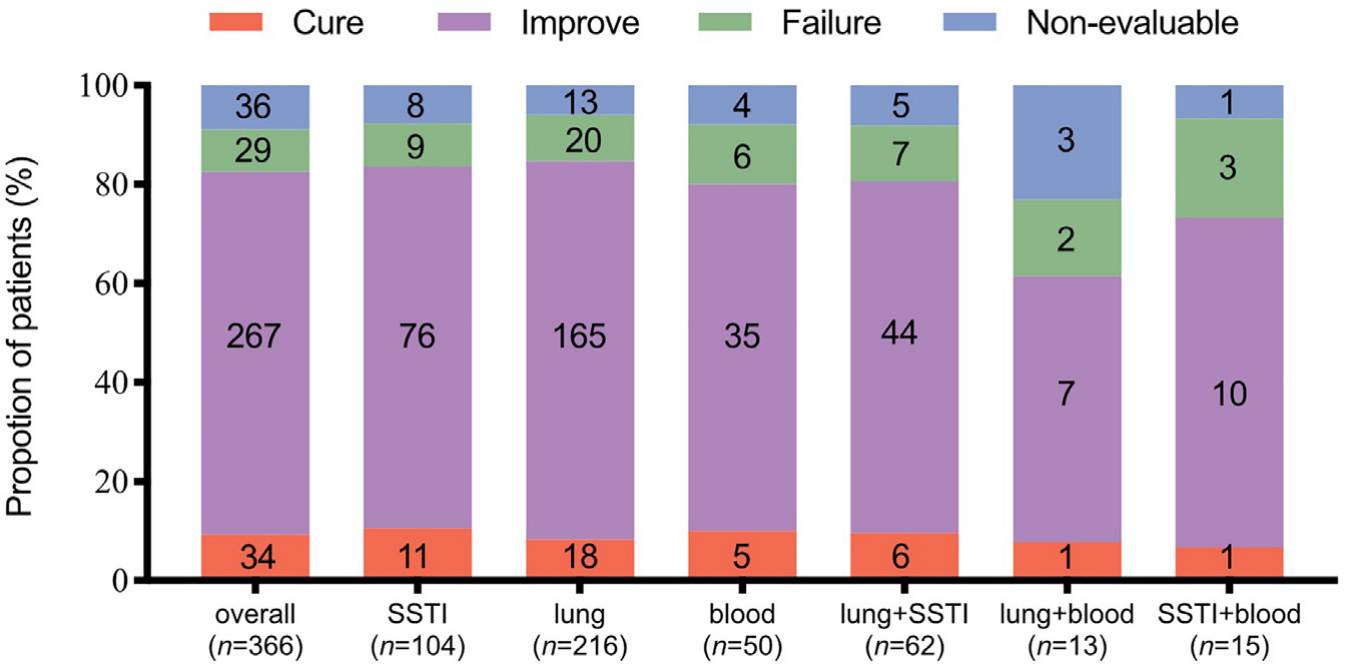
Clinical outcome by primary infection. SSTI: Skin and soft tissue infections.

Overall, clinical success was achieved in 301 (82.2%) patients; 34 (9.3%) were cured and 267 (73.0%) improved. Treatment failure was seen in 29 (7.9%) patients and a non-evaluable result was recorded in 36 (9.8%) patients. Clinical success was reported in 29 (96.7%) of 30 children; 12 (40.0%) were cured and 17 (56.7%) improved. Only one child was reported to have clinical failure. The clinical outcomes are summarized in Figure 2.

Obvious trends could be observed in patients with co-infection; the clinical failure rate was higher than with single-site infections, especially when combined with bloodstream infections. Treatment failure rates were higher in infections of the skin and soft tissue + blood (*n*=3/15, 20.0%) and lung + blood (*n*=2/13, 15.4%) than in infections of the skin and soft tissue (*n*=9/104, 8.7%), lung (*n*=20/216, 9.3%), and blood (*n*=6/50, 12.0%) alone. Clinical success rates by infection sites were similarly high in patients with SSTIs (*n*=87/104, 83.6%) and lung infections (*n*=183/216, 83.7%).

**Figure 3:**
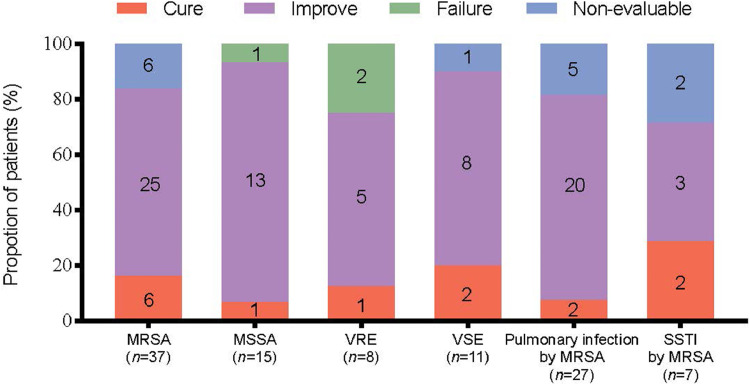
Clinical outcome by infecting pathogen. MRSA: Methicillin-resistant *Staphylococcus aureus*; MSSA: Methicillin-susceptible *Staphylococcus aureus*; SSTI: Skin and soft tissue infections; VRE: Vancomycin-resistant *Enterococci*; VSE: Vancomycin-susceptible *Enterococci*.

Treatment of MSSA infections had the highest clinical success rate (*n*=14/15, 93.3%), while that with MRSA infections came second (*n*=31/37, 83.8%). The number of enterococcal infections was limited, and the clinical success of treatment of VSE infections (*n*=10/11, 90.9%) was higher than that of VRE infections (*n*=6/8, 75%). Among the isolated MRSA-infected patients who were reclassified according to the infection site, the clinical success of treatment of MRSA pulmonary infections with linezolid (*n*=22/27, 81.5%) was higher than that of the treatment of MRSA-related SSTI (*n*=5/7, 71.5%) (Figure 3).

**Figure 4:**
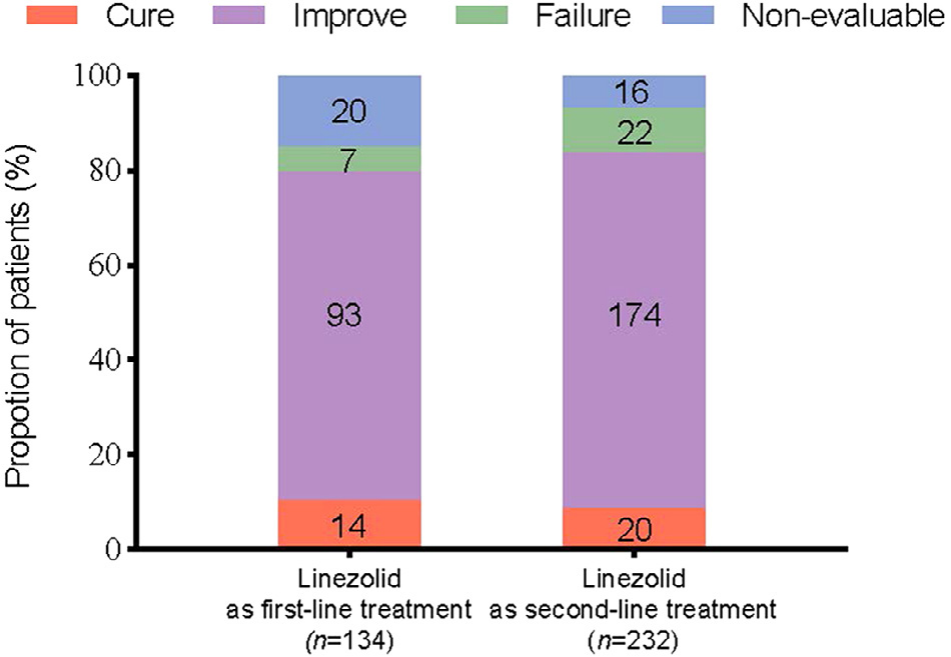
Clinical outcome by first- and second-line treatment.

Both, first-line (*n*=107/134, 79.9%) and second-line linezolid therapy (*n*=194/232, 83.6%) showed significant rates of clinical success. The failure rates in the second line (*n*=22/232, 9.5%) were higher than that of the first line (*n*=7/134, 5.2%) linezolid treatment (Figure 4).

### Safety and tolerability

Linezolid-related AEs were reported in 8 (2.2%) patients. No treatment-related SAEs were reported. A total of 3 patients experienced rash (chest rashes: *n*=2; neck rash: *n*=1), all three events were thought to be possibly related to linezolid. Overall, 2 patients were found to have lactic acidosis; one case was considered very likely to be related and the other was possibly related. A total of two patients were found to have thrombocytopenia, which was very likely to be related. Only one patient experienced possibly related acute kidney injury. Although the adverse reactions were attenuated after stopping linezolid, they persisted.

Linezolid was discontinued in 4 patients due to AEs, and there were 17 fatalities (unrelated to the study drug) throughout the trial period. There were no adverse effects recorded in children receiving linezolid therapy. Table 4 summarizes the occurrence of AEs during linezolid therapy.

**Table 4:** Treatment-related AEs of linezolid (*n=*8).

Case	Infection site	Age (years)	APACHE II	AEs	Relevance	Discontinue linezolid	Severity	Outcome
1	Lung	78	21	Acute kidney injury	Possibly related	Yes	Moderate	AE still exist
2	Abdominal	65	25	Thrombocytopenia	Very likely to be related	Yes	Mild	AE disappear
3	Lung + blood	90	30	Thrombocytopenia	Very likely to be related	Yes	Moderate	AE disappear
4	Lung	48	8	Rash (chest)	Possibly related	No	Mild	AE disappear
5	Lung	70	16	Rash (chest)	Possibly related	No	Mild	AE disappear
6	Lung	28	11	Rash (neck)	Possibly related	No	Mild	AE still exist
7	Lung	47	31	Lactic acidosis	Very likely to be related	Yes	Moderate	AE disappear
8	Lung	88	20	Lactic acidosis	Possibly related	No	Mild	AE disappear

AE: Adverse event; APACHE II: Acute physiology and chronic health evaluation.

### Linezolid prescribing patterns

The average daily dosage of linezolid was 1109.9 ± 309.9 mg. The commonly prescribed doses of linezolid in adult patients were 1200 mg (*n*=286/366, 78.1%) and 600 mg (*n*=24/366, 6.5%). In children, the commonly prescribed doses of linezolid were 1200 mg (*n*=11/30, 36.7%) and 360 mg (*n*=3/30, 10%). The mean treatment time of linezolid was 5.1 ± 4.9 days, and the total cost of linezolid therapy was ¥ 3320.9 ± 1912.7.

## Discussion

Our results provide insight into a real-world experience of linezolid use against various Gram-positive infections, including MSSA, MRSA, and VRE, in ICU patients who were severely ill and were therefore exposed to the risk of multiple nosocomial infections. Most patients included in this study were older than 65 years; patients younger than 18 years were also included. Patients were recruited from 52 hospitals in central and southwest China that had representative ICUs, enabling the analysis of a wide range of illnesses and microbiologic data. In this trial, linezolid use resulted in excellent clinical success rates in patients with Gram-positive bacterial pneumonia and SSTI. It also showed good safety and tolerability in ICU patients, including adults and children. Linezolid has shown similar high clinical success rates as both, first-line and second-line treatment; this provides more choices for clinical decision-making.

In China, despite a downward trend in recent years, multidrug-resistant Gram-positive bacteria remain one of the most important threats to human health.^[18]^ According to the results of the CHINET study, the prevalence of nosocomial drug-resistant Gram-positive infections in China is 35.3%, while that of acquired infections in the ICU is even higher.^[18]^ The low immune status of ICU patients and the use of more invasive devices such as endotracheal tubes, central venous catheters, arterial cannulas, and urinary catheters are major risk factors for Gram-positive infections. Given the correspondingly high infection burden, the precise selection of antimicrobial drugs in the ICU is crucial.[19], [20], [21]

Linezolid demonstrates significant microbial clearance for pulmonary Gram-positive bacteria, and especially MRSA, due to its characteristic high lung tissue and lung epithelial surface fluid permeability.^[22]^ In our study, linezolid showed high clinical success in the treatment of MRSA pneumonia; the clinical success rate in patients with MRSA strains isolated from the lung reached 81.5%. Vancomycin is one of the first-line treatments for MRSA pneumonia. Previous systematic reviews have shown that linezolid and vancomycin have similar clinical efficacy in MRSA pneumonia.^[^^11^^,^[23], [24], [25]^]^ Based on the above evidence, the American Thoracic Association/Infectious Diseases Association guidelines recommend vancomycin or linezolid in the treatment of MRSA pneumonia, and prefer vancomycin as the first choice.^[26]^ However, a recent meta-analysis of seven randomized controlled trials showed that linezolid-treated patients had higher clinical cure rates and microbial clearance than those treated with vancomycin.^[27]^ The findings also indicated that the treatment outcome was poor when linezolid was used after vancomycin treatment failure. In critically ill patients, prolonged endotracheal intubation is a major cause of MRSA infections. In a recent prospective observational clinical study conducted across four European ICUs,^[28]^ intravenous linezolid had better efficacy than vancomycin in reducing the burden of endotracheal MRSA in patients undergoing prolonged mechanical ventilation.

Although the number of patients with MRSA strains isolated from skin and soft tissue was limited, the results showed a high clinical success rate, particularly in terms of clinical cure. SSTIs such as impetigo, abscesses, and surgical site infections are common. Mortality and treatment costs are high for severe SSTIs, especially for those involving deep tissue.^[29]^ Linezolid and vancomycin are effective antibiotics for their treatment. A meta-analysis of nine randomized controlled trials compared linezolid with vancomycin for the treatment of SSTIs.^[30]^ Linezolid was found to be more clinically effective than vancomycin, with fewer skin complications and shorter hospital stays. More importantly, although linezolid is more expensive than vancomycin, the cost of treatment was lower than that of the latter.^[31]^

VRE is also a serious threat and has been shown to be an independent risk factor for death in patients with enterococcal bloodstream infections.^[^^32^^,^^33^^]^ Treatment options for enterococcal infections remain limited as they are highly resistant to a wide range of antibiotics. The main drugs currently available for treatment are linezolid and daptomycin. Nine previous clinical studies have shown no significant difference between daptomycin and linezolid in terms of clinical effectiveness or microbial clearance. ^[34]^ Notably, linezolid is associated with a higher risk of thrombocytopenia than daptomycin. Although linezolid was less commonly selected for patients in whom VRE strains were isolated, our results showed that it offered a good clinical success rate in VRE.

In our study, lower clinical success was observed in pneumonia or SSTIs complicated with bloodstream infections; this may be attributed to the high SOFA and APACHE II scores, which indicate a higher baseline rate of treatment failure. Findings from previous studies are consistent with our results. A 5-year retrospective study on Gram-positive bacteria isolated from bloodstream infections in critically ill patients found the SOFA score to be an independent risk factor for death in these infections.^[35]^ There was no significant difference between the three treatment regimens involving glycopeptides, linezolid, and daptomycin.

The known primary AEs associated with vancomycin use include nephrotoxicity and ototoxicity,^[^^36^^,^^37^^]^ which are especially noted in ICU patients with unstable renal function. Conversely, linezolid treatment has been linked to myelosuppression, peripheral and optic neuropathy, and lactic acidosis, especially in cases of long-term use.^[38]^ Linezolid-related myelosuppression often results in severe thrombocytopenia, which can lead to platelet transfusions, bleeding, and even an increased risk of death. The incidence of thrombocytopenia in previous studies was generally low, at approximately 2.4%; the incidence in critically ill patients and in those with low-body weight was 13.9–60.5%.^[39]^ The incidence of thrombocytopenia was low in our study and inconsistent with data from previous studies; it was observed in only two patients and the symptoms resolved after drug discontinuation. The lower incidence may have been related to the following factors: (1) linezolid use was avoided in at-risk patients (as assessed by clinicians), and (2) linezolid was only used for a short time in this study; previous studies have shown that the development of thrombocytopenia is associated with long-term use.

Linezolid is eliminated by renal (30%) and non-renal (65%) mechanisms; it is therefore less likely to cause renal injury.^[40]^ Only one patient in this cohort developed acute kidney injury; however, the renal function did not recover after discontinuation of linezolid. However, a recent meta-analysis found that linezolid use in patients with renal impairment was associated with a higher incidence of thrombocytopenia; this indicated the need for clinicians to use linezolid more cautiously in these patients.^[40]^

Despite the increasing clinical use of linezolid in adults, real-world data on linezolid use in pediatric patients is considerably limited. A total of 30 patients in this study were aged younger than 18 years. Linezolid demonstrated high efficacy and safety in the treatment of pediatric patients, with 17 children (56.7%) of clinical improved, 12 children of clinical cured (40.0%), and only 1 child of clinical failure. There were no AEs in any of the 30 patients; this suggested that linezolid can be used safely and effectively in pediatric critically ill patients. However, linezolid did not offer a significant advantage for the treatment of serious Gram-positive bacterial infections in children in previous studies; it showed neither superior nor inferior efficacy to standard antimicrobial drug therapy.^[41]^ Further qualitative studies on the use of linezolid in children are therefore needed.

## Limitations

This study has several limitations. First, this is an observational real-world study designed to realistically describe the use of linezolid in critically ill patients. It adopted broad inclusion and exclusion criteria and laid more emphasis on the actual effect in clinical patients compared with traditional randomized controlled studies. However, it could not compare the efficacy of different therapeutic drugs. Second, the sample size included in this study was insufficient; it will continue to expand while compared with other drugs in the future. Third, the first- and second-line treatments in this study referred to the first- and second-line drugs used during the period of ICU stay; the drugs used before ICU admission were not known. Fourth, the etiological evidence for Gram-positive bacteria was lower; this was mainly due to the low detection rate. Fifth, although the incidence of AEs in this study was generally consistent with those of prior phase III clinical trials, linezolid-related AEs may be subjectively underestimated by clinicians due to the complex multiple index abnormalities in ICU patients. Therefore, although linezolid showed good safety in this retrospective study, clinicians need to be cautious in evaluating possible future AEs.

## Conclusions

The treatment of Gram-positive bacterial infections in the ICU is extremely important, and the selection of appropriate antibiotics based on patients’ clinical characteristics is the key to prognosis. Based on real-world results, linezolid has been found to be effective and safe in the treatment of Gram-positive bacterial infections in critically ill patients. Linezolid showed better clinical success in pulmonary infections or SSTIs caused by *Staphylococcus aureus*. Our results will provide intensivists with a reference for the selection of medication. However, due to the limitations of this study including those pertaining to sample size, clinicians will need to individually evaluate patient conditions before using linezolid in the clinic. In addition, they will need to be vigilant regarding possible side effects during administration.

## Funding

The relevant works were supported by grants from the horizontal project of 10.13039/501100013365West China Hospital of Sichuan University (Grant No. 321190602).

## Conflicts of Interest

The authors declare that they have no known competing financial interests or personal relationships that could have appeared to influence the work reported in this paper.
